# Phylogeny and chromosomal diversification in the *Dichroplus elongatus* species group (Orthoptera, Melanoplinae)

**DOI:** 10.1371/journal.pone.0172352

**Published:** 2017-02-28

**Authors:** Elio R. D. Castillo, Alberto Taffarel, Maximiliano M. Maronna, María Marta Cigliano, Octavio M. Palacios-Gimenez, Diogo C. Cabral-de-Mello, Dardo A. Martí

**Affiliations:** 1 Laboratorio de Genética Evolutiva. Instituto de Biología Subtropical (IBS) CONICET-UNaM. FCEQyN, Félix de Azara 1552, Piso 6°. Posadas, Misiones, Argentina; 2 Comité Ejecutivo de Desarrollo e Innovación Tecnológica (CEDIT). Posadas, Misiones, Argentina; 3 Departamento de Zoologia, Instituto de Biociências, Universidade de São Paulo, Rua do Matão, Travessa 14, São Paulo, Brazil; 4 Museo de La Plata, CEPAVE, CCT La Plata, CONICET-UNLP. La Plata, Buenos Aires, Argentina; 5 UNESP—Universidade Estadual Paulista, Instituto de Biociências/IB, Departamento de Biologia, Rio Claro/SP, Brazil; Universita degli Studi di Roma La Sapienza, ITALY

## Abstract

In an attempt to track the chromosomal differentiation in the *Dichroplus elongatus* species group, we analyzed the karyotypes of four species with classical cytogenetic and mapping several multigene families through fluorescent *in situ* hybridization (FISH). We improved the taxon sampling of the *D*. *elongatus* species group adding new molecular data to infer the phylogeny of the genus and reconstruct the karyotype evolution. Our molecular analyses recovered a fully resolved tree with no evidence for the monophyly of *Dichroplus*. However, we recovered several stable clades within the genus, including the *D*. *elongatus* species group, under the different strategies of tree analyses (Maximum Parsimony and Maximum Likelihood). The chromosomal data revealed minor variation in the *D*. *elongatus* species group’s karyotypes caused by chromosome rearrangements compared to the phylogenetically related *D*. *maculipennis* species group. The karyotypes of *D*. *intermedius* and *D*. *exilis* described herein showed the standard characteristics found in most Dichroplini, 2n = 23/24, X0♂ XX♀, Fundamental number (FN) = 23/24. However, we noticed two established pericentric inversions in *D*. *intermedius* karyotype, raising the FN to 27♂/28♀. A strong variation in the heterochromatic blocks distribution was evidenced at interespecific level. The multigene families’ mapping revealed significant variation, mainly in rDNA clusters. These variations are probably caused by micro chromosomal changes, such as movement of transposable elements (TEs) and ectopic recombination. These observations suggest a high genomic dynamism for these repetitive DNA sequences in related species. The reconstruction of the chromosome character “variation in the FN” posits the FN = 23/24 as the ancestral state, and it is hypothesized that variations due to pericentric inversions has arisen independently three times in the evolutionary history of *Dichroplus*. One of these independent events occurred in the *D*. *elongatus* species group, where *D*. *intermedius* is the unique case with the highest FN described in the tribe Dichroplini.

## Introduction

The grasshopper genus *Dichroplus* Stål is dominant in South American grasslands, where the *D*. *elongatus* species group comprises representatives capable of causing considerable damage to crops and grazing [1, 2, 3]. The following eight species were included by Ronderos et al. [4] in the group, based on their similar external anatomy and body color patterns [4]: *D*. *elongatus* Giglio-Tos, *D*. *fuscus* (Thunberg), *D*. *exilis* Giglio-Tos, *D*. *patruelis* (Stål), *D*. *paraelongatus* Carbonell, *D*. *misionensis* Carbonell, *D*. *mantiqueirae* Ronderos, Carbonell & Mesa and *D*. *intermedius* Ronderos. Although recent phylogenetic hypotheses, based on a combined morphological and molecular dataset, recovered the only two representatives of the *D*. *elongatus* species group (*D*. *elongatus* and *D*. *patruelis*) included in the mentioned analysis as sister species [1, 5], the classification scheme proposed by Ronderos et al. [4] for the group has never being challenged before.

From the cytogenetic point of view, *Dichroplus* received special attention due to its chromosomal diversity. Most cases of chromosome variation in number (2n) and chromosome morphology (FN = the number of chromosome arms including the X chromosome) are recorded for the *D*. *maculipennis* species group [6]. In fact, comprehensive cytogenetic studies in this species group regarding population cytogenetics (*D*. *pratensis* Bruner) [7, 8, 9], and the structure and behavior of neo-sex chromosomes (*D*. *maculipennis* (Blanchard) and *D*. *vittatus* Bruner) [6, 10] are well known. Despite the considerable cytogenetic interest in *Dichroplus*, representatives from the *D*. *elongatus* species group have been rather neglected in this respect. Considering the amount of cytogenetic studies done in the genus, analyses of chromosome morphology and meiotic behavior are very limited for the species. The chromosome conservatism of their representatives (with 2n = 23, X0♂) could be the reason of such lack of interest in the group [11]. Current knowledge about repetitive DNA organization in *Dichroplus* chromosomes is also scarce. Concerning multigene families, the 18S, 5S rDNAs, H3 and U2 histone genes have been mapped in several related genera in the tribe Dichroplini [12, 13] but never in *Dichroplus*.

In order to contribute to the knowledge on the chromosomal differentiation pattern and their evolution, the aim of this study focuses on analyzing the chromosome morphology, structure and meiotic behavior in males and mitotic females in representatives from the *Dichroplus elongatus* species group (*D*. *elongatus*, *D fuscus*, *D*. *exilis*, *D*. *intermedius*). Moreover, we improved the taxon sampling of the species group and used molecular characters for inferring the phylogeny and hypothesize the karyotype diversification within the *D*. *elongatus* species group.

## Material and methods

### Samples

**Fig 1:**
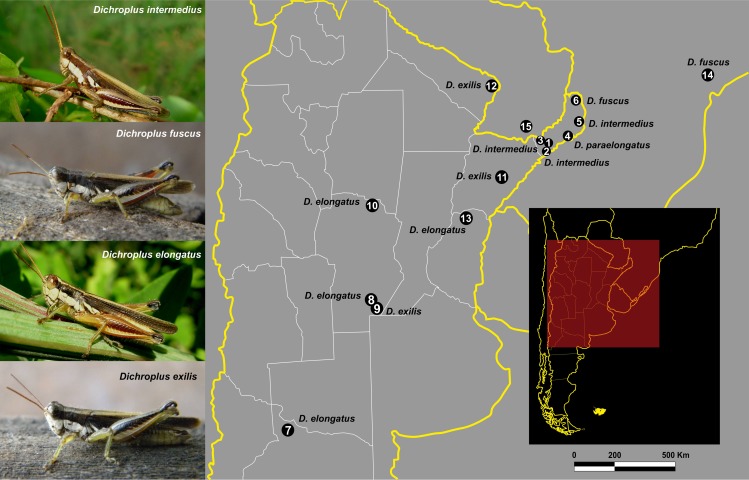
Geographic localities of *Dichroplus* grasshopper species sampled in this study. Male individuals of *D*. *intermedius*, *D*. *fuscus*, *D*. *elongatus*, and *D*. *exilis* are shown in their natural habitats. Species names in the map indicate the locality where it was sampled. Map figure reproduced from [14] under a CC BY license, with permission from (CIESIN), original copyright.

Male and female adult from the *D*. *elongatus* species group were sampled in different localities of Argentina (in Misiones with the authorization of “Ministerio de Ecología”, process number 9910-00060/13) and Brazil (in Rio Claro/SP with the authorization of COTEC process number 341/2013). The locations sampled (Fig 1) were not privately owned nor protected areas, and the field study did not involve endangered nor protected species. The information on specimens and geographic sources are provided in Table 1. Voucher specimens were deposited in the “Laboratorio de Genética Evolutiva Instituto de Biología Subtropical (IBS), CONICET-UNaM” collection.

**Table 1:** Geographic source and number of individuals per species cytogenetically analyzed in this study.

Country	Province	Locality	Lat (S) / Lon (W)	Species	Number of individuals (M/F)
Argentina	Misiones	**1.** Parada Leis	27.594092 S / 55.835036 W	*D*. *intermedius*	1/4
		**2.** Estancia La Tai Milagrosa (San José)	27.705311 S / 55.796900 W	*D*. *intermedius*	9/5
		**3.** Posadas	27.436778 S / 55.893000 W	*D*. *intermedius*, *D*. *exilis*	2/2, 1/1
		**4.** Itacaruaré	27.917500 S / 55.268556 W	*D*. *intermedius*, *D*. *paraelongatus*	3/1, 3/0
		**5.** Piñalito	26.427333 S / 53.847722 W	*D*. *intermedius*, *D*. *fuscus*	5/1, 3/4
		**6.** Cte. Andresito	25.591556 S / 53.995083 W	*D*. *fuscus*	16/14
	Rio Negro	**7.** Villa Regina	39.088667 S / 67.088000 W	*D*. *elongatus*	12/6
	Córdoba	**8.** Manantiales (Juárez Celman)	33.489972 S / 63.303111 W	*D*. *exilis*, *D*. *elongatus*	1/6, 1/1
		**9.** Estancia El Chingolo (Juárez Celman)	33.513319 S / 63.284819 W	*D*. *elongatus*, *D*. *exilis*	12/3, 7/8
		**10.** La Falda	31.089083 S / 64.456000 W	*D*. *elongatus*	1/1
	Corrientes	**11.** Paso de los Libres	29.740667 S / 57.304694 W	*D*. *exilis*	7/1
	Formosa	**12.** Palmasola	25.233444 S / 58.091583 W	*D*. *exilis*	13/3
	Entre Ríos	**13.** La Páz	30.718056 S / 59.574778 W	*D*. *elongatus*	9/12
Brazil	Sao Paulo	**14.** Rio Claro	22.396203 S / 47.538267 W	*D*. *fuscus*	6/3
Paraguay	Itapúa	**15.** Coronel Bogado	27.013750 S / 56.278222 W	*D*. *intermedius*	1/1

Country, province, locality, geographic coordinates, number of male and female individuals (M/F) per species studied.

### Chromosome and DNA samples

The insects were etherized before dissecting testis follicles and gastric caecum. Male testes were fixed in a 3:1 ethanol: acetic acid solution and female gastric caeca were removed and fixed as described by Castillo et al. [15]. All specimens were stored in 100% ethanol until subsequent DNA extraction. DNA was extracted from the hind femora of the specimens using phenol-chlorophorm procedure as described by Sambrook and Russel [16].

Male meiotic preparations were performed by squashing testes follicles in ferric hematoxylin and mitotic metaphase chromosomes from female gastric caecum were obtained following the procedure described by Castillo et al. [15]. Silver staining of kinetochores and chromatid cores were done according to the procedure of Rufas [17]. Microscopic observation of silver stained preparations involved bright field and Nomarski interference optics. C-banding was performed following the protocol of Sumner [18]. Chromomycine A_3_ (CMA_3_) and DAPI (4’, 6-diamino-2-fenilindol) staining were carried out according to Schweizer [19].

#### Isolation of multigene families and telomeric repeats

The partial sequences of 5S rDNA and histone H3 genes were obtained through Polymerase Chain Reaction (PCR), using as templates the genomic DNA of *Abracris flavolineata* (De Geer) and the primers described by Cabral de Mello et al. [20] for 5S rDNA and Colgan et al. [21] for H3 histone. The sequence for the U2 snDNA gene was obtained from *Rhammatocerus brasiliensis*´s (Bruner) genome using the primers described by Bueno et al. [22]. These sequences are deposited in GenBank (accession numbers: KC936996 for 5S rDNA, KC896792 for H3 histone gene and KC896794 for U2 snDNA). For 18S rDNA gene, a cloned fragment previously isolated from *Dichotomius semisquamosus*´s (Curtis) (Coleoptera) genome (GenBank accession number: GQ443313 [20]) was used. The telomeric motif was obtained using the self-complementary primers (TTAGG)_5_ and (CCTAA)_5_ through PCR according to Ijdo et al. [23].

#### Fluorescence in situ hybridization

The probes for 18S rDNA and H3 histone genes were labeled by nick-translation using biotin-14-dATP (Invitrogen, San Diego, CA, USA), while the 5S rDNA, U2 snDNA and telomeric probes sequences were labeled through PCR with digoxigenin-11-dUTP (Roche, Mannheim, Germany). Single and two color FISH experiments were performed as in Cabral-de-Mello et al. [20]. Probes labeled with digoxigenin-11-dUTP were detected using anti-digoxigenin-rhodamine (Roche), and probes labeled with biotin-14-dATP were identified using streptavidin, alexafluor 488 conjugate (Invitrogen). The preparations were counterstained using DAPI and mounted using Vectashield (Vector, Burlingame, CA, USA). The FISH results were documented using an Olympus microscope BX61 equipped with a fluorescence lamp and appropriate filters coupled to DP70 cooled digital camera. The images were merged and optimized for brightness and contrast using Adobe Photoshop CS2 software.

### Phylogenetic analyses

#### Molecular data set

Taking into account the previous analysis on our target group (*Dichroplus elongatus* species group) and related genera performed by Colombo et al. [1], we selected the same molecular markers for our phylogenetic inference: mitochondrial genes cytochrome oxidase I (COI) and cytochrome oxidase II (COII). In order to complement the already existing data (COI and COII sequences), we provided newly-generated sequence data for five members of *D*. *elongatus* species group (*D*. *intermedius*, *D*. *fuscus*, *D*. *exilis*, *D*. *paraelongatus*, *D*. *elongatus*; see Table 2 for details). Additionally, new-molecular sequences data were generated for the related species *B*. *punctulatus*, *Scotussa cliens*, *D*. *maculipennis* and *D*. *vittatus* (see Table 2 for detail).

**Table 2:** List of species analyzed including ID, collecting event (country, province, town, date) and accession numbers for COI and COII.

Species name	Specimen ID	Locality and date	COI	COII
*Apacris rubritorax*	GenBank		AF539848 a	AF539846 a
*Baeacris punctulatus*	**DM7115**	Argentina, Misiones, Candelaria. IV-28-14	**KY595083**	**KY595074**
*Baeacris pseudopunctulatus*	GenBank	Argentina, Buenos Aires Trenque Lauquen. I-09-99	DQ083452	DQ083436
*Atrachelacris unicolor*	GenBank	Argentina, Misiones, Concepción. I-09-14	AY014360	AY014358
*Atrachelacris olivaceus*	GenBank	Argentina, Córdoba, Capilla del Monte. II-14-00	DQ083451	DQ083435
*Ronderosia bergii*	GenBank	Argentina, Buenos Aires, Pehuajo. II-02-98	DQ083467	DQ083448
*Ronderosia forcipata*	GenBank	Argentina, Buenos Aires, Pehuajo. II-11-98	DQ083468	DQ083449
*Scotussa impúdica*	**DM 6771**	Argentina, Misiones, Concepción. I-09-14	**KY595091**	**KY595082**
*Scotussa lemniscata*	GenBank	Argentina, Buenos Aires, Benito Juárez II-15-01	DQ 389229	DQ 389215
*Scotussa daguerrei*	GenBank	Argentina, Buenos Aires, Benito Juárez. I-05-00	DQ083469	DQ083450
*Leiotettix sanguineus*	GenBank	Argentina, San Luis, Buena Esperanza. II-15-01	DQ083465	DQ083446
*Leiotettix pulcher*	GenBank	Argentina, Buenos Aires, Pehuajó. I-27-02	DQ083464	_
*Leiotettix viridis*	GenBank		AY014353a	_
*Neopedies brunneri*	GenBank	Argentina, San Luis, Merlo. II-27-01	DQ083466	DQ083447
*Neopedies noroestensis*	GenBank		AF539852 a	AF539850 a
*Pseudoscopas nigrigena*	GenBank		AY014349 a	AY014347 a
*Dichroplus maculipennis*	**DM3279**	Argentina, Buenos Aires, Benito Juárez. II-26-10	**KY595088**	**KY595079**
*Dichroplus conspersus*	GenBank	Argentina, Buenos Aires, Pigüe II-15-00	DQ083454	DQ083438
*Dichroplus vittatus*	**DM1923**	Argentina, Río Negro, Villa Regina. IV-17-09	**KY595090**	**KY595081**
*Dichroplus vittigerum*	GenBank	Argentina, Rio Negro, Bariloche I-19-02	DQ083463	DQ083445
*Dichroplus democraticus*	GenBank	Argentina, Rio Negro, Bariloche I-30-02	DQ083455	_
*Dichroplus schulzi*	GenBank	Argentina, Formosa, Las Lomitas IV-04-99	DQ083460	DQ083443
*Dichroplus pratensis*	GenBank	Argentina, La Pampa, Santa Rosa I-09-99	DQ083458	DQ083442
*Dichroplus patruelis*	GenBank	Argentina, Buenos Aires, Benito Juárez I-21-02	DQ083458	DQ083441
*Dichroplus obscurus*	GenBank	Argentina, Buenos Aires, Benito Juárez I-08-02	DQ083457	DQ083440
*Dichroplus silveiraguidoi*	GenBank	Uruguay. II-2-02	DQ083461	_
*Dichroplus elongatus*	GenBank		AF260551 b	AF260549 b
*Dichroplus elongatus* (LGE)	**DM 4682**	Argentina, Córdoba, La Falda. II-25-12	**KY595084**	**KY595076**
*Dichroplus exilis*	**DM 2405**	Argentina, Misiones, Posadas. I-11-10	**KY595085**	**KY595077**
*Dichroplus paraelongatus*	**DM 2886**	Argentina, Misiones, San Javier. III-05-10	**KY595089**	**KY595080**
*Dichroplus intermedius*	**DM 3019**	Paraguay, Coronel Bogado. II-21-10	**KY595087**	**KY595075**
*Dichroplus fuscus*	**DM 3532**	Argentina, Cte. Andresito, Misiones. III-16-10	**KY595086**	**KY595078**

Technical details on DNA extraction, PCR profiles, primers, and sequencing reactions can be found in Colombo et al. [1] and Litzenberger and Chapco [24], which we followed. For each gene, sequences were assembled and aligned in Geneious R8 [25] considering invertebrate mitochondrial translation code (using Translation Align tool), and thereafter concatenated dataset from these two gene alignments were conducted in SequenceMatrix 1.7.9. [26]. The newly generated sequences were deposited in GenBank (accession numbers are given in Table 2).

#### Maximum Parsimony (MP)

Phylogenetic analyses of the molecular matrix were performed under MP using the software TNT v1.1 [27]. The data set was analyzed using unweighted standard parsimony [28]. The heuristic search procedure consisted of "TBR branch swapping" applied to a series of 500 random addition sequences, retaining ten trees per replicate.

#### Maximum Likelihood (ML)

The concatenated dataset was partitioned according to molecular markers to estimate the best models of nucleotide substitution for each partition using jModelTest 2 [29] (Dataset (terminals) partition + ModelTest: COI (32 terminals) 633 bp, COII (29 terminals) 357 bp + BIC: COI = TIM2+I+G, COII = TrN+G). ML phylogenetic analyses were conducted in IQ-TREE1.4.2 [30]; (total of 50 individual replicates, considering 500 initial parsimony trees and all possible nni movements). Parametric (abayes, alrt) and non-parametric (traditional bootstrap -500 replicates- and sh-alrt -5000 replicates) support methods were computed in IQ-TREE as well [31]. Alternative analytic scenarios were generated, including best-fit partition scheme (PartitionFinder 2.0 [32]) and filtered potential non-phylogenetic genetic information using Aliscore (default parameters [33]); in all cases phylogenetic inferences were calculated with IQ-TREE as detailed above (see S1 Table for a summary and comparison with main ML result).

### Karyotype optimization

To evaluate the *D*. *elongatus* species group karyotype evolution onto the *Dichroplus* phylogeny, we mapped the character “variation in the fundamental number (FN)” using the software Mesquite Version 3.10 under default parameters in MP and ML. We considered karyotype information about the fundamental number (FN) of the species analyzed in this work and from publications where authors proposed a hypothesis about the origin of the chromosome number [7, 8, 34, 35].

Character states for the “variation in the FN” were arbitrarily coded as 0: FN = (no variation in the number of chromosome arms FN = 23/24); 1: (increasing in the number of chromosome arms due to two fixed pericentric inversions FN = 27/28); 2: (reduction in the number of chromosome arms due to centric fusion, pericentric inversions FN = 19/20); 3: (reduction in the number of chromosome arms due to a complex karyotype origin FN = 12/13). State 2 was coded following the hypothesis of Saez and Perez-Mosquera [34, 35], which proposed the origin of *D*. *pratensis* karyotype through two centric fusions (involving four non-homologous acrocentric chromosomes) and two pericentric inversions (reducing the ancestral number of chromosome arms from 23⁄24 to 19⁄20). *Dichroplus silveiraguidoi* was arbitrarily coded as 3 following the same criteria used in Colombo et al. [1]: there is no evidence about the number of fusions that could have taken place during the evolution of this species; there is no other species with an intermediate state of karyotype reduction; the different states (0–3) mapped on the tree are unordered and do not constitute a transformation series.

## Results

### Karyotypes and heterochromatin

**Fig 2:**
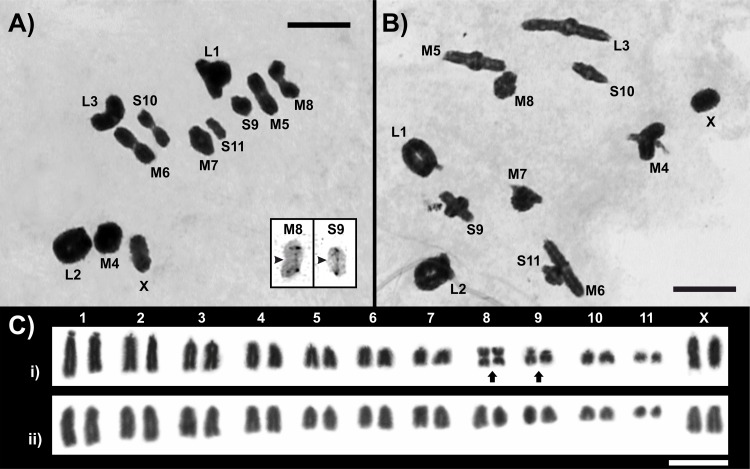
Male meiosis and female mitosis of *Dichroplus intermedius* and *D*. *exilis*. (A) Male metaphase I, showing eleven autosomal bivalents and the X chromosome of *Dichroplus intermedius*, inset showing the M8 and S9 pair with a failed chiasma; (B) Male metaphase I, showing eleven autosomal bivalents and the X chromosome of *D*. *exilis*; (C) Karyotypes of female mitotic metaphases from gastric caecum i) *D*. *intermedius*, the metacentric M8 and a submetacentric S9 pairs indicated with black arrows, ii) *D*. *exilis* showing 22 telocentric autosomal pairs and two telocentric X chromosomes. Bar = 10 μm.

The karyotypes of *D*. *intermedius* and *D*. *exilis* are described for the first time herein. Both species showed 2n = 23 and a X0 sex chromosome determination system (males) and the chromosomes were arranged in three large (L1-L3), five medium (M4-M8), and three small (S9-S11) bivalents, plus the X chromosome which size is similar to M4 chromosome (Fig 2A and 2C). In *D*. *intermedius* we observed 20 telocentric, two metacentric and two submetacentric chromosomes, pairs M8 and S9 respectively (Fig 2Ci), while in *D*. *exilis* all chromosomes were telocentric (Fig 2Cii). The FN observed in male meiotic cells of *D*. *intermedius* and *D*. *exilis* was 27 and 23 respectively, while female mitotic cells showed 28 in *D*. *intermedius* and 24 in *D*. *exilis*. At metaphase I, both M8 and S9 pairs showed two configurations in *D*. *intermedius*: about 87% of the cells showed the metacentric M8 chromosomes with a distal chiasma per arm (n = 230), although in some cases failed chiasma was evidenced (13%). Besides, S9 pair failed a chiasma in the short arm, evidenced by a C-shape configuration at metaphase I, through the inter-chromatidic chores structures (n = 230, 22%) (Fig 2A inset).

*Dichroplus elongatus* is characterized by 2n = 23, X0 in males [36], while *D*. *fuscus* exhibited a variation in the 2n from 22–23, X0 in males due to the presence of a heterozygous Robertsonian fusion (Rb-fusion) [37].

**Fig 3:**
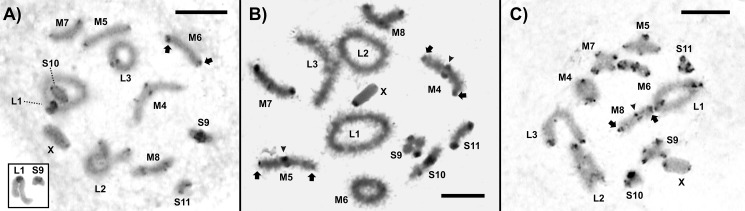
C-banding. Male diplotene. (A) *Dichroplus intermedius*, arrow showing the centromeric heterochromatin; the inset shows the proximal heterochromatic block in L1 and S9. (B) *Dichroplus exilis*, centromeric (arrow) and distal (arrow heads) heterochromatic blocks are indicated in M4-M5. (C) *Dichroplus elongatus*, arrow and arrow heads indicate centromeric and distal heterochromatin. Bar = 10 μm.

**Fig 4:**
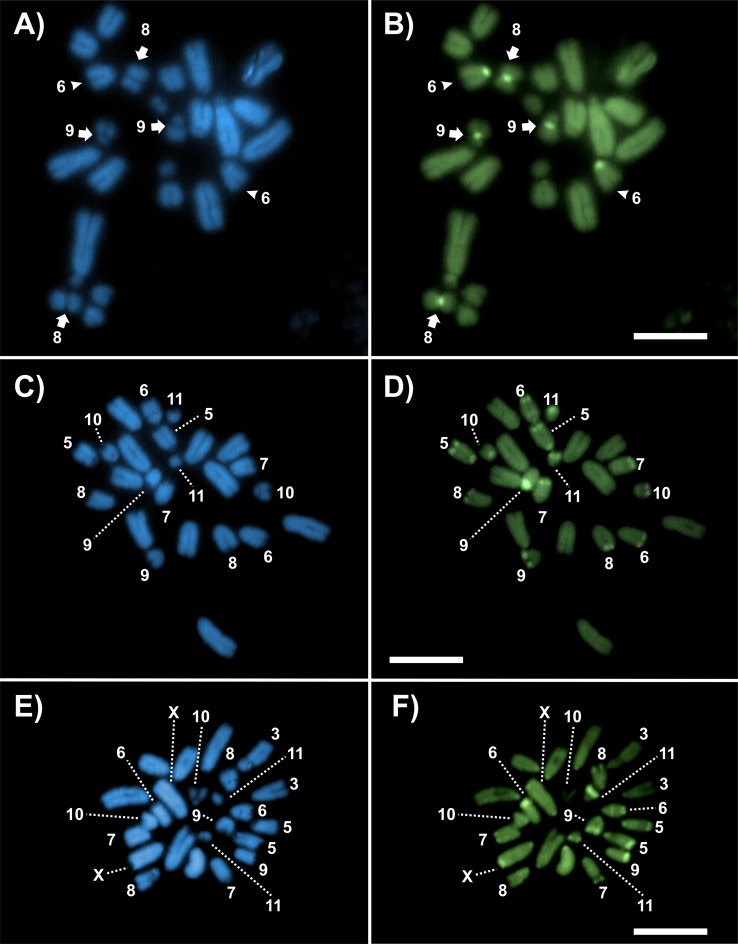
CMA_3_ fluorochrome staining. Female mitotic metaphases from gastric caecum; (A-B) *D*. *intemedius*, CMA_3_+/DAPI^_^ centromeric blocks are indicated in M6 with arrow heads and with arrows in M8 and S9. (C-D). *D*. *exilis* and (E-F) *D*. *fuscus*, centromeric and distal blocks CMA_3_+/DAPI^_^ are indicated in the autosomes and the X chromosomes. Bar = 10 μm.

The C-banding analysis in male meiosis of *D*. *intermedius* revealed C-positive blocks in the centromeric region of the entire set (Fig 3A), with conspicuous proximal heterochromatic blocks in pairs L1 and S9 (Fig 3A, inset). In *D*. *exilis*, C-positive pericentromeric blocks along the entire complement were observed (Fig 3B). Fluorescent staining using CMA_3_ and DAPI for *D*. *intermedius* revealed CMA_3_+ and DAPI^_^ signals in the pericentromeric regions of M6, M8 and S9 pairs (Fig 4A and 4B). *Dichroplus exilis* presented CMA_3_+ and DAPI^_^ signals in the centromeric and distal region of M5 pair, the distal region of M7, M8, S9, S11 pairs and the pericentromeric region of M6 (Fig 4C and 4D). In *D*. *elongatus* we observed a centromeric and telomeric pattern of heterochromatin distribution (Fig 3C) and evidenced a similar fluorochrome banding pattern through the sequential CMA_3_/DAPI staining described by Rosetti et al. [36]. *Dichroplus fuscus* presented C-positive blocks in the centromeric region of the entire chromosome set; bivalents M5-M8 and S9-S11 also showed C-positive heterochromatic blocks in their telomeric regions, as well as the X chromosome. Sequential CMA_3_/DAPI banding revealed CMA_3_+/DAPI^_^ bands in centromeric regions; terminal CMA_3_+/DAPI^_^ bands were brighter in M5, M6, S9 and S11 pairs. Besides, the X chromosome showed CMA_3_+ signals in the prericentromeric and distal regions. The pericentromeric block in pair M3 was negative for both, DAPI and CMA_3_; an interstitial band in chromosome M6 was CMA_3_+/DAPI^_^ (Fig 4E and 4F).

### Multigene families and telomeres mapping

**Fig 5:**
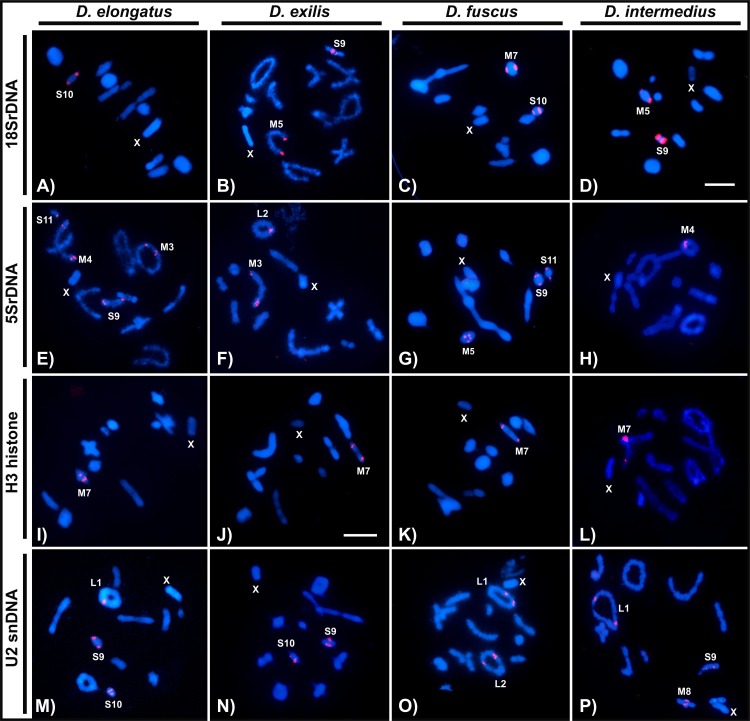
FISH with 18S, 5S rDNA, H3 histone and U2 snDNA probes in meiotic cells from males. The probe and species name are indicated in each figure. Chromosomes with positive signals and the X chromosome are indicated. (A-D) 18S rDNA, (E-H) 5S rDNA, (I-L) H3 histone gene and (M-P) U2 snDNA. Bar = 10 μm.

**Fig 6:**
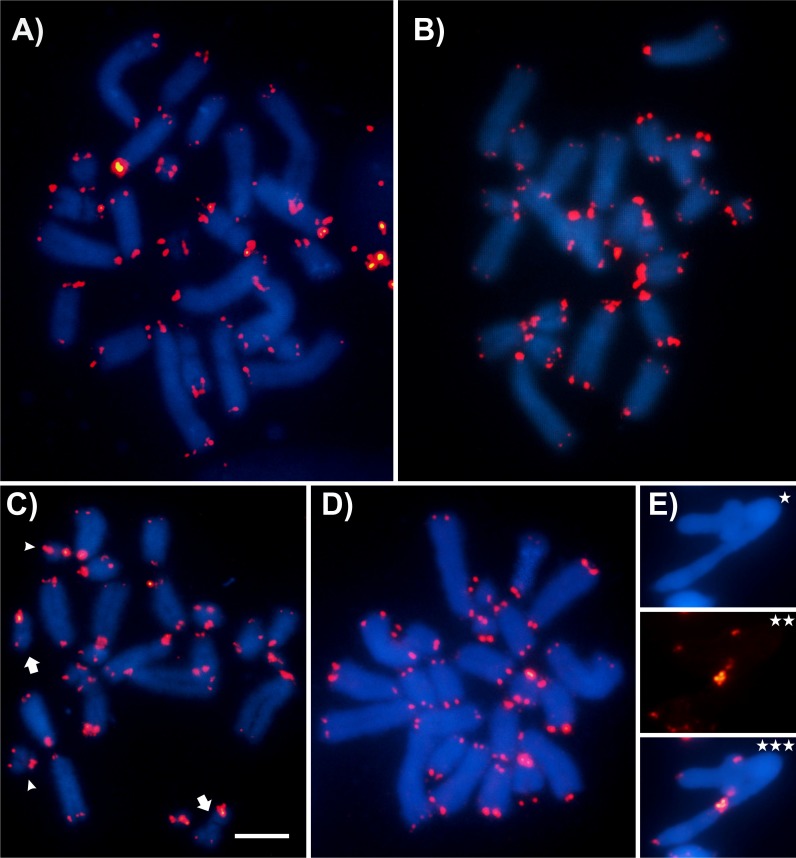
**FISH with telomeric probe in female gastric caecum (A, B, C, D) and male meiotic cells (E).** (A) *D*. *elongatus*; (B) *D*. *exilis*; (C) *D*. *intermedius*, arrow and arrow heads indicate M8 and S9 autosome pairs; (D, E) *D*. *fuscus*, (E) *DAPI, **probe, *** overlapping. Bar = 10 μm.

**Fig 7:**
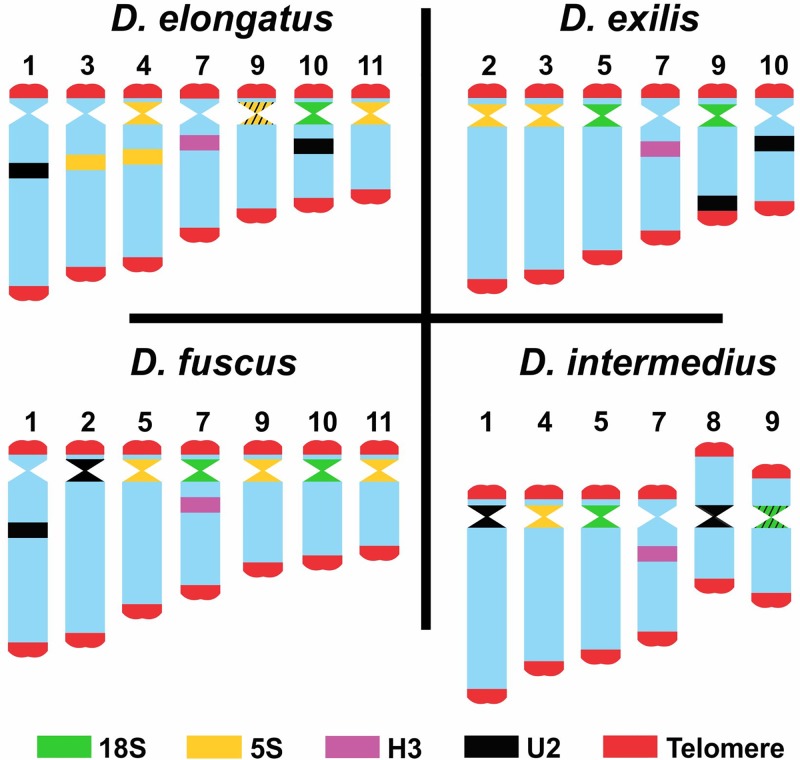
Ideogram showing FISH signals of all the chromosomal markers analyzed for the *Dichroplus* species. The probes and their relative position on each chromosome are indicated using colors. Only chromosomes with markers are shown.

FISH analysis with the distinct probes revealed variable patterns depending on the sequence mapped. The 18S rDNA was invariably located in pericentromeric region of distinct chromosomes (one or two bivalents), depending on the species (Fig 5A–5D, Table 3), while the 5S rDNA although frequently placed in pericentromeric region, was also observed in interstitial clusters in *D*. *elongatus* (Fig 5E–5H, Table 3). The number of clusters for 5S rDNA was variable in the four species, ranging from two clusters (one bivalent) to eight clusters (four bivalents) (Fig 5E–5H, Table 3). The unique conserved cluster, regarding number and position, was the H3 histone, which was placed in the interstitial position, but not far from the centromere, in the M7 pair (Fig 5I–5L, Table 3). The U2 snDNA was observed mainly in the largest autosomal pair, a pattern that was observed in three species and additional clusters in other chromosomes were also noticed (Fig 5M–5P, Table 3). Finally, the telomeric probe revealed signals only in the terminal regions observed in female mitosis (Fig 6A, 6B, 6C and 6D). It was noted the absence of interstitial telomeric sites in M8-S9 autosomes of *D*. *intermedius*, yield by pericentric inversions (Fig 6C) and also in *D*. *fuscus* metacentric autosome, observed in meiosis, produced by a Rb-fusion (Fig 6E). Fig 7 summarizes the markers obtained with FISH where each chromosome can be differentially recognized by morphology, size, presence/absence and position of the markers.

**Table 3:** Summary of chromosomal data including diploid numbers, Fundamental Number (FN) and chromosomal locations of multigene families for each species analyzed in this study.

Species	2n(M/F)	FN	18S rDNA	5S rDNA	H3 Histone gene	U2 snDNA
*D*. *elongatus*	23/24	23/24	S10pc	M3i; M4pc, i; S9pc; S11pc	M7i	L1i; S9pc; S10i
*D*. *exilis*	23/24	23/24	M5pc; S9pc	L2pc; M3pc	M7i	S9d; S10i
*D*. *fuscus*	23/24	23/24	M7pc; S10pc	M5pc; S9pc; S11pc;	M7i	L1i; L2pc
*D*. *intermedius*	23/24	27/28	M5pc; S9pc	M4pc	M7i	L1pc; M8pc; S9pc

2n: diploid number; FN: fundamental number; M: male; F: female; pc: pericentromeric; i: interstitial; d: distal.

### Phylogenetic relationships of the *D*. *elongatus* species group and karyotype character optimization

**Fig 8:**
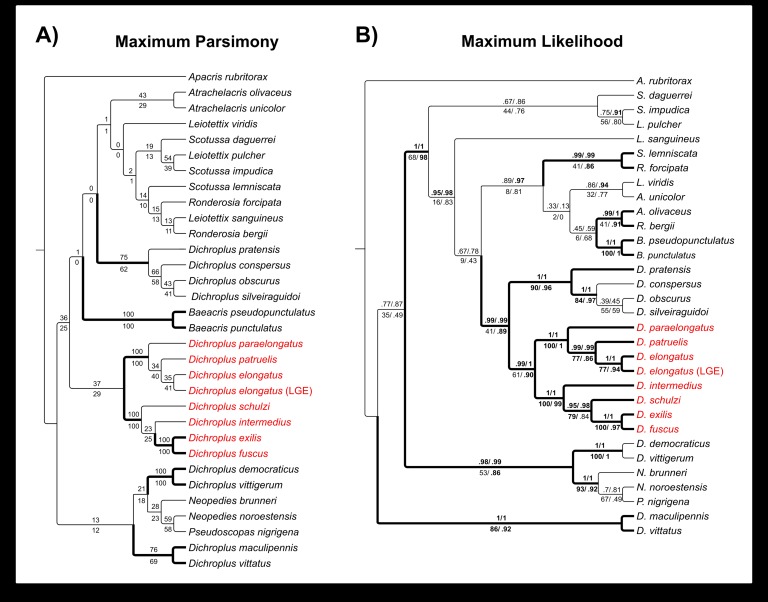
**Phylogenetic trees obtained from the concatenated datasets (A) Maximum Parsimony (MP) tree and (B) Maximum Likelihood (ML) tree**. Support values are integer numbers or decimals. In MP tree, number above branches is the resampling value and below branches is the bootstrap value; in ML tree, the number above the branches (aBAYES/aLRT) and non-parametic support below the branches (BS/SH-aLRT). Values in plain text indicate non-significant support (aBAYES< 0.95; aLRT< 0.9; BS < 75%; SH-aLRT< 0.85); significant support values are in bold (aBAYES ≥ 0.95; aLRT ≥ 0.9; BS ≥ 75%; SH-aLRT ≥ 0.85). Thick lines indicate significant support in trees obtained in at least 3 out of 4 searching strategies; thin black lines indicate low support in trees obtained from two or fewer methods. The *D*. *elongatus* species group clade is shown in red.

**Fig 9:**
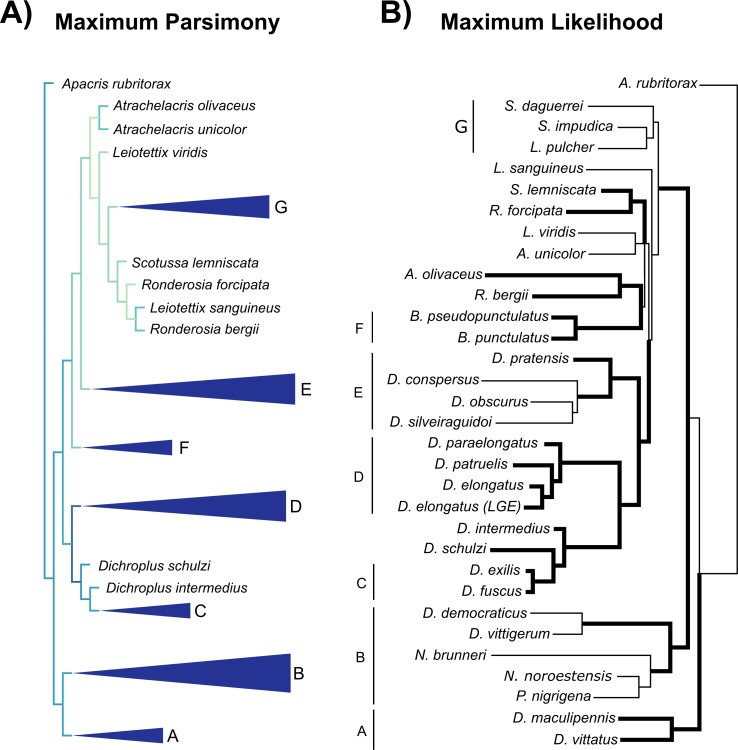
Side-by-side comparison of the Maximum parsimony (MP) and Maximum Likelihood (ML) trees. (A) MP tree showing congruent nodes as collapsed for comparison; colors in tree branches represent similarity with ML tree (from light color–none or low similarity- to heavy blue–total similarity [38]). (B) ML tree, format of lines as used in Fig 8; numbered groups indicate clades with congruent groupings in MP result.

The molecular analysis performed employing the MP criteria recovered a fully resolved tree (L = 1150; CI = 48; RI = 50; Fig 8) but with no evidence or support for the monophyly of *Dichroplus*, in agreement with the hypothesis proposed by Colombo et al. [1] and our ML results (Fig 8 and S1 Fig). Representatives of the *D*. *elongatus* species group resolved as monophyletic if *D*. *schulzi* is included in the group. Most analyses recovered three groups well supported: (*D*. *schulzi* (*D*. *intermedius* (*D*. *fuscus*, *D*. *exilis*))), (*D*. *paraelongatus* (*D*. *patruelis*, *D*. *elongatus*)) and (*D*. *pratensis* (*D*. *conspersus* (*D*. *silveiraguidoi*, *D*. *obscurus*))) (Fig 9). The main difference between MP and ML is the relationship of the *D*. *elongatus* species group to the other species; in the ML tree topology the clade was recovered as a sister group of the clade (*D*. *pratensis* (*D*. *silveiraguidoi* (*D*. *obscurus*, *D*. *conspersus*)) whereas in the MP tree it was recovered as a sister group of ((*B*. *pseudopunctulatus*, *B*. *punctulatus*) (*D*. *pratensis* (*D*. *conspersus* (*D*. *silveiraguidoi*, *D*. *obscurus*))) ((*A*. *olivaceus*, *A*. *unicolor*) ((*L*. *viridis* (*S*. *daguerrei* (*L*. *pulcher*, *S*. *impudica*))) (*S*. *lemniscata* (*R*. *forcipata* (*L*. *sanguineus*, *R*. *bergii*))))). With the exception of *Baeacris* (two species sampled), none of the remaining sampled genera were recovered as monophyletic (Figs 8 and 9).

**Fig 10:**
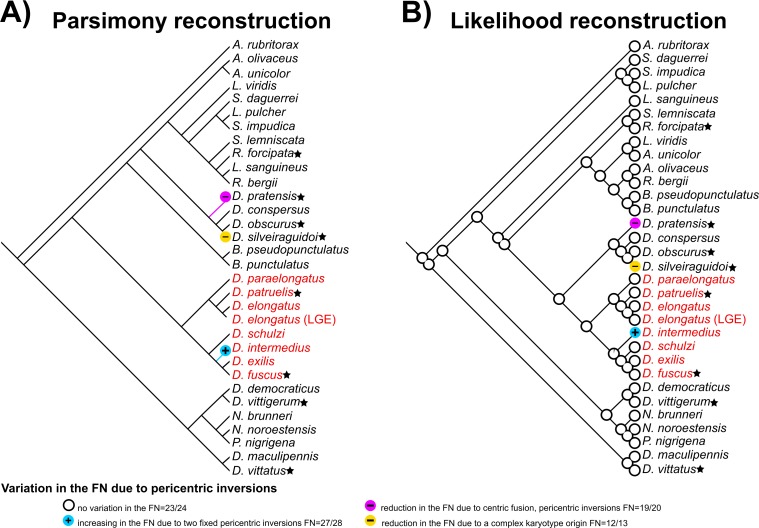
**Ancestral character state reconstruction on the (A) MP (unordered) and (B) ML (categorical Mk1 model) trees**. The *D*. *elongatus* species group clade is shown in red. Minus symbol represent hypothetical reduced ancestral number of chromosome arms (FN) for *D*. *pratensis* and *D*. *silveiraguidoi*, and plus symbol represent hypothetical increased number of chromosome arms (FN) for *D*. *intermedius*. Black stars represent information about reduction in the chromosome number due to A-A centric fusions placed next to each terminal into the MP and ML tree.

Ancestral reconstruction of the chromosome character “variation in the FN” onto the MP and ML phylogenies is shown in Fig 10. According to the optimization of the karyotype in the tree, variations in the number of chromosome arms (FN), due to pericentric inversions, arose independently three times: in *D*. *intermedius*, *D*. *pratensis* and *D*. *silveiraguidoi*. Besides, the optimization on both trees suggested the FN = 23/24 as the ancestral state for the group.

## Discussion

Species of *D*. *elongatus* group analyzed here shared several taxonomic characters [4, 39], and at the chromosomal level show slight variations due to the occurrence of chromosome rearrangements compared with the *D*. *maculipennis* species group [6, 40]. Despite the standard male chromosome number (2n = 23) observed in the species analyzed here, we noticed a diversification pattern concerning the multigene family genes, probably caused by micro chromosomal rearrangements that led to the divergence of the chromosomal markers employed in this work. In the molecular phylogeny presented here, the *D*. *elongatus* species group resolved as monophyletic if *D*. *schulzi* is included in the group, and the internal relationships recovered for the group are in agreement with the scheme proposed by Ronderos et al. [4].

Considering several chromosomal aspects described in *Dichroplus* (i.e. diploid number, fundamental number, sex chromosome system), the *D*. *elongatus* species group showed five of eight representatives (i.e. *D*. *exilis*, *D*. *paraelongatus*, *D*. *misionensis*, *D*. *mantiqueirae* and *D*. *elongatus*) with the standard chromosome number of most Acrididae grasshoppers 2n = 23/24, X0/XX, FN = 23/24 [1, 40]. While a standard chromosome number was also observed in *D*. *intermedius*, it showed an increase in the FN, due to an established pericentric inversion, evidenced by the occurrence of two metacentric (pair M8) and two submetacentric (pairs S9) chromosomes. At least at the tribe level (Dichroplini), *D*. *intermedius* is the first case with a radical increase in the FN. The group also presents species (i.e. *D*. *patruelis* and *D*. *fuscus*) with a reduction in chromosome number, although the fundamental number remains constant [36, 37, 40, 41].

The chromosome stability of the *D*. *elongatus* species group becomes evident when it is contrasted with the *D*. *maculipennis* species group. Most *Dichroplus* species with modified karyotypes are included in the *D*. *maculipennis* species group, with six species showing neo-XY sex chromosome systems and one case of a complex polymorphic system [1, 6, 40]. While standard karyotypes (2n = 23/24, X0/XX, FN = 23/24) have been described in two species, it is not the rule [1, 6].

Karyotype variation due to pericentric inversions, as detected in *D*. *intermedius*, it is not a common feature in acridids, and even much less common in South American Melanoplinae, but isolated cases were reported in others subfamilies (e.g. South American Ommexechinae [42, 43]; Gomphocerinae, *Sinipta dalmani* (Stål) [44]; Oedipodinae, *Trimerotropis* spp [45]). In other Dichroplini species (*Dichroplus vittatus* Bruner, *Ronderosia bergii* (Stål) and *Boliviacris noroestensis* Ronderos & Cigliano), pericentric inversions possibly played an important role in the origin and differentiation of neo-sex chromosome systems [6, 46, 47]. Moreover, in the case of two other *Dichroplus* species, *D*. *pratensis* and *D*. *silveiraguidoi*, pericentric inversions together with centric fusions proved to be a parsimonious explanation for the origin of their karyotypes, reducing the 2n and the number of chromosome arms (FN) [11, 35]. These data suggest that pericentric inversions are relevant forces driving the diversification of karyotypes in *Dichroplus*.

### Chromosomal organization and diversification patterns in standard karyotypes

Although species with standard karyotypes analyzed here showed a similar pattern of heterochromatin distribution to those found in most Melanoplines (heterochromatin localized in the pericentromeric region) [11], the evidence provided here noted strong variations among karyotypes of the *D*. *elongatus* species group (Fig 3). Interspecific variations were observed where the most variable pattern concerning size and block locations was evidenced in *D*. *elongatus* (Fig 3C). C-banding pattern noticed in the species group led us to propose the hypothesis that intraespecific difference could be an indicator that heterochromatin rearrangements might have a role in the *D*. *elongatus* species group’s karyotypic evolution [36]. The results observed for CMA_3_/DAPI support this idea; the information provided in this work indicates a significant degree of chromosome differentiation at interspecific level, when the distribution pattern and composition of heterochromatin are considered (Fig 4).

Although six out of eight species from the *D*. *elongatus* species group show the standard chromosome number 2n = 23/24, our results revealed by FISH pointed out a differential interspecific chromosome pattern for the number of rDNA clusters not leaded by obvious macro chromosomal changes. Based on our observations, a high genomic dynamism for these rDNA sequences is evident in phylogenetically related species. In this sense, intra and intergenomic variability for the multigene families were observed concerning the mapped sequences in *Dichroplus* species. Our findings are consistent with previous studies in grasshopper species, where a remarkable variability in the number and location of major rDNA genes were observed [48]. Similar patterns were evidenced in other groups of insects (i.e Lepidoptera [49], Coleoptera [50] and Triatominae Heteroptera [51]), which are caused by micro and macro chromosomal rearrangements. A similar situation was also noticed in the species study here, with high variation for the number of sites for 5S rDNA clusters. Thus, *D*. *intermedius* carry it in a single chromosome pair and *D*. *elongatus* in four pairs, with intermediate patterns in the remaining species analyzed. In Acrididae species from the old world, the pattern found showed an extensive variation in number and sites of 5S rDNA, which included variability at intraspecific level as in *Eyprepocnemis plorans* (Charpentier) [12, 52]. Like for major rDNA (45S), the movement and multiplication of 5S rDNA could be mostly attributed to micro chromosomal changes. Both sites of rDNA (45S, 5S) are a common target of TEs [53, 54, 55, 56], which could facilitate the colonization process in different chromosomes. Variability for U2 snDNA was also noticed, but the frequent presence of this marker in the pair 1 in Melanoplinae grasshoppers [12, 13] suggests that it could be the modal pattern, being the other sites caused by amplification and transposition events. Additionally, this marker changed position in the pair 1 from interstitial in *D*. *fuscus* and *D*. *elongatus* to the proximal region in *D*. *intemedius*, suggesting the possible occurrence of paracentric inversion in this chromosome, or intrachromosomal movement. Among other Orthoptera species, including *Abracris flavolineata* (grasshopper) and *Cycloptiloides americanus* (Saussure) (cricket) this gene was also placed in the pair 1, but in other animal groups like fish, the occurrence of distinct patterns with scattered organization and multiple sites were reported, as we also noticed it here [57, 58].

An opposite pattern to those seen for rDNAs and U2 snDNA was evidenced for the H3 histone gene, which was placed in a single chromosome in the species analyzed (pair 7). It resembles the most common chromosomal pattern (chromosome number and position) for this gene in grasshoppers, i.e. one interstitial cluster per haploid genome in most species [59]. The only difference is related to the specific chromosome bearing this marker, which frequently is the 8th in size rank order. A possible explanation to the observed difference could be assigned to those caused by loss or gain of chromatin due to repetitive DNAs in these medium sized chromosomes in the *D*. *elongatus* species group, being the transposition events less probable, although the dispersion of this marker was also noticed [12, 13, 22, 60].

Our results obtained related to the absence of an internal telomeric sequence (ITS) (Fig 6) suggest that the breakage for this rearrangement occurred near to the centromeric region, not involving the telomere. Like in *D*. *intermedius*, *D*. *fuscus* populations from Rio Claro/SP (Brazil) (with a reduction in the diploid number 2n = 22 caused by a chromosomal fusion in heterozygosis between pairs 1/3) did not reveal ITS, also suggesting loss of telomere during the rearrangement. Although in both cases a posterior loss of telomeric motif after the rearrangement could not be completely ruled out or the occurrence of small number of repetition not detected by classical FISH technique.

The results presented here are congruent with the pattern found in South American Melanoplinae [12] supporting the high genomic dynamism for these repetitive DNA sequences observed in the analyzed *Dichroplus* species. Although they showed a different level of dispersion, the comparative analysis with species published by Palacios-Gimenez et al. [12] (*Dichromatos schrottkyi* (Rehn), *Dichromatos lilloanus* (Liebermann), *Eurotettix minor* Bruner, *Eurotettix brevicerci*, *Chlorus chiquitensis* Cigliano & Lange, *Chlorus vittatus* Bruner) shows a frequent location (i.e. pericentromeric, never distal) for the 18S rDNA in the *Dichroplus* species analyzed here. The presence of several clusters of 5S rDNA in different locations reported in Palacios-Gimenez et al. [12] revealed less interspecific variation of this marker compared with our results. While, in phylogenetically related genera of South American Melanoplinae (*Dichromatos*, *Eurotettix*, *Chlorus*), several clusters of U2 snDNA were noticed in different positions [12], together with the information provided in this study, we could infer a shared pattern in the interstitial position in pair 1. Based on the available information, the H3 histone gene mapped in several representatives of Acrididae and Proscopiidae showed a restrict location to a single autosomal pair [60, 61]. The conservative interstitial position in pair 7 found in all the representatives from the *D*. *elongatus* species group analyzed in this work could indicate a shared location at least at the genus level.

### Karyotype evolution of the *D*. *elongatus* species group in the phylogenetic context of *Dichroplus*

Based on our phylogenetic results, the four species of the *D*. *elongatus* species group added to the *Dichroplus* phylogeny were placed in a single branch together with *D*. *schulzi*, in agreement with the relationships found by Colombo et al. [1] and Dinghi et al. [5]. In these hypotheses, the authors recovered *D*. *elongatus* and *D*. *patruelis* together with *D*. *schulzi* in the molecular and combined trees. Our analyses showed *D*. *schulzi* related to *D*. *intermedius*, *D*. *fuscus* and *D*. *exilis* strongly supported when different approaches were conducted (Fig 8). Besides, nonparametric and parametric strategies followed in this work provided congruent results concerning the relationships among the remaining representatives from the *D*. *elongatus* species group (Figs 8 and 9). Despite doubts expressed regarding the inclusion of *D*. *schulzi* within the genus *Dichroplus* [62] the results of our molecular data analyses, reinforce with additional evidence the affinity proposed previously between *D*. *schulzi* and the *D*. *elongatus* species group [1, 5], especially with *D*. *intermedus*, *D*. *exilis* and *D*. *fuscus*.

The molecular phylogeny presented in this work showed stable and well supported relationships of the *Dichroplus elongatus* species group, and congruent results between the different strategies implemented. Numerous studies on the importance of dense taxon sampling have indicated that introducing additional taxa into a phylogenetic analysis results in more accurate estimates of evolutionary relationships [63]. Indeed, our results suggest that both taxon and data sampling efforts will enhance future phylogenetic analysis, and eventually a new classification scheme for the group [63, 64]. In this sense, improved taxon sampling allowed us not only to infer the *D*. *elongatus* species group relationships but also to reconstruct chromosomal evolution onto a robust phylogeny. Thereby, optimization of the chromosome character “variation in the FN” onto the *Dichroplus* phylogeny showed that variation due to pericentric inversions in *D*. *intermedius* (Fig 10, blue circle with plus symbol), *D*. *pratensis* (Fig 10, purple circle with minus symbol) and *D*. *silveiraguidoi* (Fig 10, yellow circle with minus symbol) arose independently in *Dichroplus*, where the only case of an increased FN within the genus and at the tribe level, was evidenced in *D*. *intermedius* karyotype.

It is important to note that Autosome-Autosome (A-A) centric fusions also played a role in the chromosomal evolution in *Dichroplus* [1, 6, 10, 37, 40, 65, 66]. In this sense, we interpreted the character “reduction in the chromosome number due to A-A centric fusions” in the MP and ML tree, with a different criterion to those used in previous work [1], and considered it as independent events. This interpretation was made because autosomes from the standard karyotype involved in the rearrangement are difficult to determine and as a consequence homologous A-A fusions could not be established. Thus, cases of reduction in the chromosome numbers due to A-A centric fusions occurred repeatedly and independently in *Dichroplus*, described in *D*. *pratensis* (complex system of polymorphic centric fusions), *D*. *obscurus* (two homozygous A-A centric fusion), *D*. *vittigerum* (two homozygous A-A centric fusion), *D*. *vittatus* (one telocentric A-A centric fusion) and *D*. *silveiraguidoi* (several A-A centric fusions) (Fig 10, black stars). Concerning the *D*. *elongatus* species group, reduction in the chromosome number through A-A centric fusion could have arisen twice, once in *D*. *patruelis* (one fixed A-A centric fusion) and another time in *D*. *fuscus*, as a complex system of polymorphic centric fusions (Fig 10, black stars) [37, 40].

The results presented in this work provide relevant information about karyotype evolution in the *D*. *elongatus* species group within a molecular phylogenetic hypothesis of *Dichroplus*, leaving open future studies. It is worth noting that further analyses involving all the species of the genus should be conducted to test the monophyly of *Dichroplus*, employing multiple character sources. Moreover, the results presented here, under the molecular cytogenetics framework, provided an initial characterization of multigenes family in *Dichroplus* species; to obtain a more detailed picture of the chromosomal diversification and the evolutionary dynamics of multigene families at this level, future studies involving other *Dichroplus* species should be performed.

## Supporting information

S1 Fig Phylogenetic tree considering alternative ML results. Clade stability for main result considering presence in alternative ML results (relative percentage and ML strategy as follows: 1: Partition Finder partition; 2: no partition + jModelTest; 3: Alicore + no partition + ModelTest; 4: Alicore + partition + ModelTest; 5: PartitionFinder k-means). Example: branch (*Scotussa lemniscata*, *Ronderosia forcipata*) is represented as well in 80% of alternative ML analysis strategies (trees from 1, 2, 3 and 4). All alternative results were estimated with same basic parameters in IQ-TREE as in main ML result. See main text and S1 Table for details on data and applied software.
(TIF)

S1 Table Alternative 5 ML analysis, detailing partition, model selection and filtering strategies (first column); total number of bases per partition and analysis (second column); and evolutionary model definition with Bayes Information Criteria (third column). Used software for model selection: PartitionFinder [32] and jModelTest 2 [29]; Aliscore [33] was used for filtering potential noisy data. See S1 Fig for results’ comparison with main ML result.
(DOCX)
