# Supplementary figures and images for: Phylogeny and chromosomal diversification in the Dichroplus elongatus species group (Orthoptera, Melanoplinae)
Source: PLoS One. 2017 Feb 28;12(2):e0172352. doi: 10.1371/journal.pone.0172352 (PMC5330476; doi:10.1371/journal.pone.0172352)

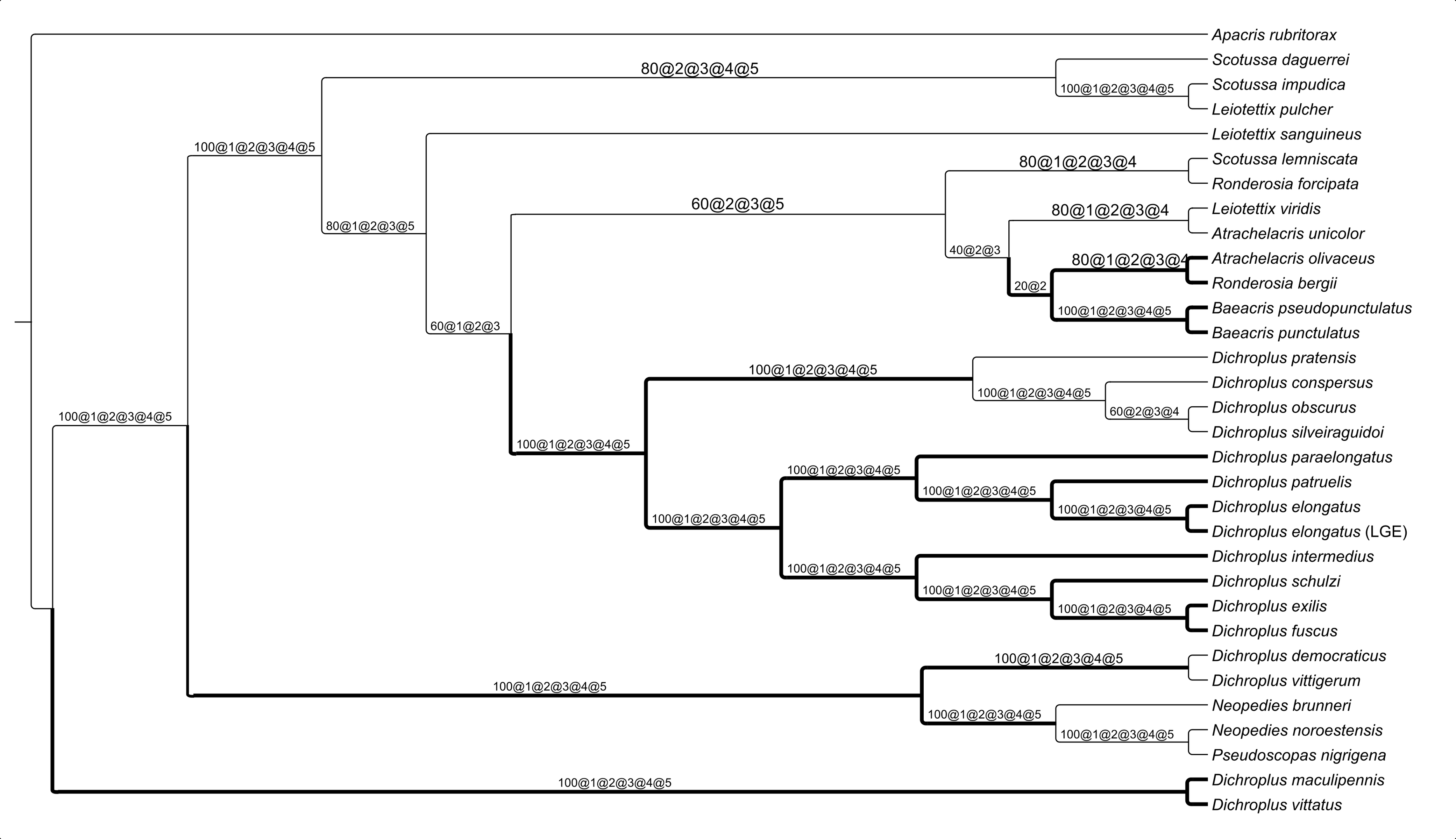

Supplement: S1 Fig — Clade stability for main result considering presence in alternative ML results (relative percentage and ML strategy as follows: 1: Partition Finder partition; 2: no partition + jModelTest; 3: Alicore + no partition + ModelTest; 4: Alicore + partition + ModelTest; 5: PartitionFinder k-means). Example: branch (Scotussa lemniscata, Ronderosia forcipata) is represented as well in 80% of alternative ML analysis strategies (trees from 1, 2, 3 and 4). All alternative results were estimated with same basic parameters in IQ-TREE as in main ML result. See main text and S1 Table for details on data and applied software. (TIF) [file pone.0172352.s001.tif]
