# Supplementary material for: Phylogeny and chromosomal diversification in the Dichroplus elongatus species group (Orthoptera, Melanoplinae)
Source: PLoS One. 2017 Feb 28;12(2):e0172352. doi: 10.1371/journal.pone.0172352 (PMC5330476; doi:10.1371/journal.pone.0172352)
Supplement: S1 Table — Used software for model selection: PartitionFinder [32] and jModelTest 2 [29]; Aliscore [33] was used for filtering potential noisy data. See S1 Fig for results’ comparison with main ML result. (DOCX) [file pone.0172352.s002.docx]

| **Strategy** | **Dataset (bp)** | **Evolutionary model** |
| --- | --- | --- |
| **1**: PartitionFinder partition + model definition | Bases | BIC |
| COI, COII 1 position | 330 | TIMef+I+G |
| COI, COII 2 position | 330 | F81+G |
| COI, COII 3 position | 330 | TVM+G |
| **2**: No partition + jModelTest | Bases | BIC |
| COI and COII datasets | 990 | TIM2+I+G |
| **3**: Filter + no partition + jModelTest | Bases | BIC |
| COI and COII | 852 | TIM2+I+G |
| **4**: Filter + partition + jModelTest; | Bases | BIC |
| COI | 620 | TIM2+I+G |
| COII | 343 | TrN+G |
| **5**: Aliscore + PartitionFinder k-means partition | Bases (total:852) | BIC |
| Partition 1 | 170 | TIMEF |
| Partition 2 | 510 | F81 |
| Partition 3 | 172 | TIM+G |
